# Vitamin B-6, Independent of Homocysteine, Is a Significant Factor in Relation to Inflammatory Responses for Chronic Kidney Disease and Hemodialysis Patients

**DOI:** 10.1155/2017/7367831

**Published:** 2017-09-26

**Authors:** Cheng-Hsu Chen, En-Ling Yeh, Chih-Chung Chen, Shih-Chien Huang, Yi-Chia Huang

**Affiliations:** ^1^Division of Nephrology, Department of Internal Medicine, Taichung Veterans General Hospital, Taichung 40705, Taiwan; ^2^Division of Basic Medical Sciences, Department of Medical Research, Taichung Veterans General Hospital, Taichung 40705, Taiwan; ^3^Department of Life Science, Tunghai University, Taichung 40705, Taiwan; ^4^Graduate Program in Nutrition, Chung Shan Medical University, Taichung 40201, Taiwan; ^5^Division of Nephrology, Department of Internal Medicine, Chiayi Branch, Taichung Veterans General Hospital, Chiayi 60090, Taiwan; ^6^Department of Nutrition, Chung Shan Medical University, Taichung 40201, Taiwan; ^7^Department of Nutrition, Chung Shan Medical University Hospital, Taichung 40201, Taiwan

## Abstract

The purpose of this study was to investigate whether plasma pyridoxal 5′-phosphate (PLP) and homocysteine were dependent on or independent of each other in order to be associated with inflammatory markers in patients with chronic kidney disease (CKD) or those receiving hemodialysis treatment. This was a cross-sectional study. Sixty-eight stage 2–5 CKD patients and 68 hemodialysis patients had one time fasting blood drawn for measurements of plasma PLP, pyridoxal (PL), homocysteine, and several inflammatory markers. Early CKD stage (stages 2-3) patients showed significantly lower plasma PLP levels and homocysteine concentrations than patients in an advanced CKD stage (stages 4-5) and those undergoing hemodialysis. Plasma PLP significantly correlated with CRP levels (partial *r*_*s*_ = −0.21, *p* < 0.05) and plasma PL significantly correlated with IL-10 levels (partial *r*_*s*_ = −0.24, *p* < 0.01), while plasma PLP plus PL significantly correlated with both CRP levels (partial *r*_*s*_ = −0.20, *p* < 0.05) and interleukin-1*β* (partial *r*_*s*_ = 0.22, *p* < 0.05) levels after adjusting for plasma homocysteine and other potential confounders. Plasma homocysteine displayed no significant correlations with any inflammatory markers. Vitamin B-6 status, rather than homocysteine, appeared to be a significant factor in relation to inflammatory responses for CKD and hemodialysis patients.

## 1. Introduction

Patients with chronic kidney disease (CKD) or receiving hemodialysis treatment are under persistent low-grade inflammatory status [1–6] and have been suggested to experience poorer clinical conditions and higher mortality when they had persistent elevated levels of C-reactive protein (CRP), interleukin-6 (IL-6), interleukin-8 (IL-8), or tumor necrosis factor-*α* (TNF-*α*) [7–9]. Many factors may be associated with inflammation for CKD and hemodialysis patients; however, its causes have not been fully elucidated.

Low vitamin B-6 status [10–12] and hyperhomocysteinemia [13–15] commonly exist in patients with CKD or those receiving hemodialysis treatment, and these two factors could be implicated in inflammatory responses. Plasma pyridoxal 5′-phosphate (PLP, an active form of vitamin B-6) possibly acts as a coenzyme for the production of cytokines and other polypeptide mediators during the inflammatory responses [16]. The relationship between low vitamin B-6 levels and levels of inflammatory markers has been observed in patients with inflammatory diseases [17–20] and even independently of plasma homocysteine in the population-based Framingham Heart cohort study [16]. However, no association between plasma PLP and cytokine levels [i.e., IL-6, interleukin-10 (IL-10) and transforming growth factor-*β*] was found in kidney transplant recipients [21, 22]. In addition to the relationship between vitamin B-6 and inflammation, a high homocysteine concentration was also significantly correlated with inflammatory markers (i.e., CRP, IL-6, IL-8, and TNF-*α*) among the elderly and patients with cardiovascular disease [23–26]. Homocysteine is oxidized to form a disulfide bond with another homocysteine or protein when it is released from the cell into the blood circulation via a superoxide free radical which is released at the same time [24]. This can then give rise to oxidative and nitrosative stress and initiate inflammation via the regulation of NF-*κ*B transcription [27, 28]. Although homocysteine can be used as an inflammatory marker to indicate the presence of inflammation, increased homocysteine concentration and elevated inflammatory markers were not always associated with each other [24]. Since the discrepancy between vitamin B-6 or homocysteine and inflammation still exists, the roles vitamin B-6 and homocysteine status play in the inflammatory responses need to be further confirmed.

Plasma PLP is an essential coenzyme of transsulfuration in homocysteine metabolism, so a low or deficient PLP may thus cause the accumulation of homocysteine. If CKD patients or hemodialysis patients were potentially under persistent low-grade inflammation and had low vitamin B-6 levels and a high homocysteine status, questions would be raised to whether there were associations among vitamin B-6, homocysteine concentrations, and inflammatory markers and whether vitamin B-6 and homocysteine were dependent on or independent of each other when associated with inflammatory markers. Therefore, the purpose of this study was to investigate the dependent or independent association of plasma PLP and homocysteine with inflammatory markers in both CKD and hemodialysis patients.

## 2. Subjects and Methods

### 2.1. Study Design and Sample Size Calculation

This was a cross-sectional study. A previous study indicated that plasma PLP was significantly associated with CRP (partial *r* = −0.25, *p* < 0.05) [20]. Thus we needed a sample size of 124 subjects based on a power of 80% and a 2-sided test with *α* of 0.05 to detect a significant correlation coefficient of 0.25 between plasma PLP and CRP levels.

### 2.2. Subjects

Patients fell within the ages of 20 to 85 years and either were with stage 2–5 CKD [estimated glomerular filtration (eGFR) ≤ 89 mL/min/1.73 m^2^] or were receiving hemodialysis treatment with high-flux polysulfone membrane dialyzers (FX 80® or FX 100®) operated by Fresenius 4008S dialysis systems (Fresenius Medical Care AG & Co., Bad Homburg, Germany) for 4 hours three times per week. They were recruited from the outpatient clinic and the hemodialysis center of the Division of Nephrology of Taichung Veterans General Hospital, Taiwan. Patients' diagnoses and CKD staging were confirmed by an experienced nephrologist. Exclusion criteria included patients who were clinically unstable, pregnant, lactating, or having a history of liver disease, chronic inflammatory disease, cancer, or alcoholism. This study was approved by the Institutional Review Board of Taichung Veterans General Hospital (IRB TCVGH numbers SF13223 and SF15019A). Participants provided their informed consent prior to enter the study.

### 2.3. Data Collection and Biochemical Measurements

Subjects' age, gender, smoking, and drinking habits, along with their nutritional supplements were recorded. CKD patients' height, weight, waist circumference, and systolic and diastolic blood pressure (SBP and DBP) were measured on an appointed day while patients receiving hemodialysis treatment were measured on an appointed day at a prehemodialysis session. Body mass index (BMI, kg/m^2^) was then calculated.

Blood samples taken after a period of fasting were drawn and collected in vacutainer tubes (Becton Dickinson, Franklin Lakes, NJ, USA) containing ethylenediaminetetraacetic acid as the anticoagulant or with no anticoagulant on an appointed day for CKD patients and on an appointed day (the second dialysis session of the week) at a prehemodialysis session for hemodialysis patients. Serum or plasma was separated within 30 minutes after blood was collected and immediately measured or then frozen (−80°C) until analysis. Serum albumin, glucose, total cholesterol, creatinine, and blood urea nitrogen were measured using an automated biochemical analyzer. Automated high-sensitivity CRP measurement concentration was performed using particle-enhanced immunonephelometry with an image analyzer. Plasma PLP, pyridoxal (PL), and homocysteine were quantified by high performance liquid chromatography using fluorescence detection following the methods of Talwar et al. [29] and Araki and Sako [30]. Vitamin B-6 deficiency was defined as a plasma PLP level < 20 nmol/L [31]. Hyperhomocysteinemia was defined as a plasma homocysteine concentration ≥ 14 *μ*mol/L [32]. Homocysteine and vitamin B-6 measurements were carried out under yellow light to prevent photodestruction. Different plasma cytokine levels [i.e., interleukin-1*β* (IL-1*β*), interleukin-4 (IL-4), IL-6, IL-8, IL-10, and TNF-*α*] were analyzed by using a magnetic bead-based multiplex immunoassay system (Bio-Plex) (BIO-RAD Laboratories, UK) following the manufacturer's instructions. All analyses were performed in duplicate.

### 2.4. Statistical Analyses

All data analyses were performed by using the SAS statistical software package (version 9.3; Statistical Analysis System Institute Inc., Cary, NC, USA). A Shapiro-Wilk test was performed to test the normal distribution. Differences in patients' anthropometric measurements and biochemical values were compared for significant differences amongst the groups and analyzed by a one-way analysis of variance or Kruskal-Wallis one-way analysis of variance on ranks. For categorical response variables, differences amongst the groups were assessed by the Chi-square test. A partial Spearman correlation coefficient (*r*_*s*_) was used to analyze the correlations of plasma PLP, PL, and homocysteine with inflammatory markers after adjusting for the potential confounders. Results were considered statistically significant at *p* < 0.05. Values presented in the text are means ± standard error (SE).

## 3. Results

Of sixty-eight total patients, 13 had stage 2 (eGFR, 60–89 mL/min/1.73 m^2^), 21 had stage 3 (eGFR, 30–59 mL/min/1.73 m^2^), 21 had stage 4 (eGFR, 15–29 mL/min/1.73 m^2^), and 13 had stage 5 (eGFR, <15 mL/min/1.73 m^2^) CKD. The main etiologies of CKD were diabetic nephrology, IgA nephrology, gouty nephrology, nephrotic syndrome, or tubulointerstitial nephritis. An additional 68 patients receiving hemodialysis treatments were also recruited for this study. Patients had been dialyzed for 7.63 ± 7.82 year. There were similar results between stages 2 and 3, and stages 4 and 5 (data not shown). We then pooled stage 2 and 3 CKD patients (*n* = 34) into an early CKD stage group (eGFR, 33.15–88.04 mL/min/1.73 m^2^; median eGFR, 53.63 mL/min/1.73 m^2^) and along with pooling stage 4 and 5 patients (*n* = 34) into an advanced CKD stage group (eGFR, 6.97–29.13 mL/min/1.73 m^2^; median eGFR, 17.88 mL/min/1.73 m^2^) based on the clinical description of CKD stages in order to increase the studies statistical strength.

Patients receiving hemodialysis treatment were the oldest in age and had a lowest eGFR level and the highest SBP, serum creatinine, and BUN levels, while patients in the early CKD stage were the youngest and had the highest eGFR level and the lowest SBP, serum creatinine, and BUN levels amongst the 3 groups (Table 1). There were no significant differences in BMI, DBP, and serum glucose levels among groups (Table 1). Only one hemodialysis patient had a BMI < 18.5 indicating underweight. More than one-third of patients receiving hemodialysis treatments were taking B-vitamin supplements, while no early CKD stage patients had taken B-vitamin (folate, vitamin B-6, and vitamin B-12) supplements (Table 1).

Table 2 shows vitamin B-6 and homocysteine concentrations, along with levels of inflammatory markers in the 3 groups. Early CKD stage patients displayed significantly lower plasma PLP and homocysteine concentrations than the patients who were receiving hemodialysis treatments. However, there was no significant difference in plasma PL concentration among the 3 groups. Only 10 patients had a vitamin B-6 deficiency (plasma PLP level < 20 nmol/L). There was a 91.2% prevalence of hyperhomocysteinemia (plasma homocysteine concentration ≥ 14 *μ*mol/L) in our patients. Since B-vitamin supplement use may affect homocysteine concentration, we further compared the plasma homocysteine concentration between patients who were taking or not taking B-vitamin supplements within the advanced CKD stage and hemodialysis group. Patients who were taking B-vitamin supplements had a significantly lower plasma homocysteine concentration than patients who were not taking B-vitamin supplements (22.93 ± 3.08 vs. 29.36 ± 1.74 *μ*mol/L) in the advanced CKD stage group. Hemodialysis patients who were taking B-vitamin supplements had only slightly, not significantly, lower plasma homocysteine concentration than patients who were not taking B-vitamin supplements (25.75 ± 1.18 versus 28.34 ± 1.66 *μ*mol/L). There were no significant differences in IL-4, IL-6, and IL-8 levels among the 3 groups, while CRP, IL-1*β*, IL-10, and TNF-*α* levels remained significantly different among the 3 groups.

We excluded 39 patients who were taking B-vitamin supplementation in order to understand the association between vitamin B-6 status and inflammatory markers without the confounding of B-vitamin supplement use. The associations were similar between vitamin B-6 status and inflammatory markers with and without including patients who were taking B-vitamin supplementation (data not shown). We thus presented the results for all patients (*n* = 136), adjusting B-vitamin supplement use during partial Spearman correlation coefficient analyses. Plasma PLP significantly correlated with CRP levels, while plasma PL significantly correlated with IL-10 level and plasma PLP + PL significantly correlated with CRP and IL-1*β* levels after adjusting for age, gender, BMI, SBP, serum albumin concentration, diabetes, CKD stage, B-vitamin supplement use, smoking, and drinking habits and finally additionally adjusting for plasma homocysteine concentration (Table 3). There was no association found between plasma PLP and homocysteine concentration, and plasma homocysteine concentration was not correlated with any inflammatory markers after adjusting for potential confounders (data not shown).

## 4. Discussion

To the best of our knowledge, the relationship between vitamin B-6 status, plasma homocysteine, and inflammatory markers has not been simultaneously or extensively investigated in CKD and hemodialysis patients. Cytokines are released from inflammatory cells and drive the inflammatory process. For example, IL-1*β*, has a strong proinflammatory role; IL-6 and TNF-*α* are active contributors to acute inflammatory responses. We thus assessed plasma PLP, PL, and homocysteine concentrations, as well as different cytokine markers in our CKD and hemodialysis patients.

In the vitamin B-6 metabolism, PLP is dephosphorylated by alkaline phosphatase to PL before entering the cell, where the PL is then reformed to PLP by pyridoxine kinase. Since the interconversion between PLP and PL is reversible and widely distributed, plasma PLP + PL can thus be considered as a single pool of vitamin B-6 [20]. We then combined plasma PLP and PL and observed that the plasma PLP + PL correlated with CRP and IL-1*β*. In accord with the previous study [20], plasma PLP + PL as well as individual plasma PLP or plasma PL could indicate that vitamin B-6 catabolism is increased during inflammation. We recommend that plasma PLP and PL be simultaneously assessed in CKD and hemodialysis patients.

CRP is produced in the liver and its level rises rapidly after an inflammatory stimulus. We excluded clinically unstable CKD and hemodialysis patients, and our patients were regularly checked up by the nephrologist and had the stable disease condition which may not cause too severe inflammatory responses; it might be the reason for our patients had substantially lower CRP levels when compared to the data of patients from the National Health and Nutrition Examination Survey III (NHANS III) [1] and other studies [9]. However, our advanced CKD stage and hemodialysis patients had higher CRP levels than early CKD stage patients. The association between low plasma PLP and high CRP levels not only was observed in patients with inflammatory diseases [17–20] but also was presented in patients with type 2 diabetic nephrology [33], along with patients with CKD and those undergoing hemodialysis in the present study. In contrast with Jankowska et al. [21], an indication of no relationship between plasma PLP or PL and inflammatory markers was due to the lack of vitamin B-6 deficiency in their kidney allograft recipients. Although 7.4% of our patients had plasma PLP < 20 nmol/L and 23.5% of our patients (particularly those in advanced CKD stage and those undergoing hemodialysis) were taking vitamin B-6 supplements which led to the mean plasma PLP level having an adequate status (>20 nmol/L) and levels in a large variation, significant associations between vitamin B-6 and CRP, IL-1*β*, and IL-10 existed in our CKD and hemodialysis patients. Even though patients who were taking B-vitamin supplements were excluded in order to reduce the variation of plasma PLP and PL, we still observed the significant associations between vitamin B-6 status and inflammatory markers in our patients. Paul et al. [34] revealed out that the negative association between plasma PLP and inflammatory responses was not truly linked to vitamin B-6 deficiency but was rather associated with a metabolic phenomenon inherent to inflammation. Zhang et al. [35] indicated that higher physiological concentrations of PLP and PL are needed to inhibit both Toll-like receptor-induced NF-*γ*B activation and NLRP3-mediated caspase-1 activation to eradicate IL-1*β* production in macrophages [35]. It seems that a higher vitamin B-6 status is required for CKD and hemodialysis patients to cope with their inflammatory responses. In addition, a higher need for vitamin B-6 may not be mediated through homocysteine, since plasma homocysteine was additionally adjusted in the correlation analyses and there was no correlation seen between plasma PLP and homocysteine. Other mechanisms may also be involved in vitamin B-6 and inflammation. Vitamin B-6 could be stemming from the mobilization of plasma PLP to an inflammatory site for degradation of tryptophan via the kynurenine pathway, metabolism of immunomodulatory sphingolipids, and the proliferation of immune cells [16, 34, 36].

Circulating inflammatory markers, such as IL-6, IL-8, and TNF-*α*, have been shown to be associated with the loss of renal function [4], while IL-6 and TNF-*α* have been considered as good inflammatory markers for risk stratification in CKD [3, 6, 7, 37]. Although both IL-6 and TNF-*α* stimulate CRP production, we only observed that vitamin B-6 status correlated with CRP but did not correlate with IL-4, IL-6, IL-8, and TNF-*α*. IL-6 and TNF-*α* are proinflammatory mediators and systemically increase rapidly (~1.5–3 hours) while CRP levels increase slightly later (~6–8 hours) following tissue injury [8]; therefore a large interindividual variation for these inflammatory markers (IL-6 and TNF-*α*) may be a possible reason for there being no significant associations of vitamin B-6 with these inflammatory markers in the present study.

One of the major causes of hyperhomocysteinemia might be due to loss of renal function [15, 38, 39]. In concordance with previous studies [40–43], hyperhomocysteinemia was prevalent in our CKD and hemodialysis patients, and homocysteine levels were 2-fold higher in hemodialysis patients than those in early stage CKD in the present study. However, unlike previous studies [23–26, 41], hyperhomocysteinemia did not contribute to inflammatory responses in our CKD and hemodialysis patients. There was also no significant association shown between homocysteine and CRP in ischemic stroke patients [44] or homocysteine and IL-6 levels in the general population [45]. Although hyperhomocysteinemia has been implicated as an important risk factor of inflammatory responses [23–26, 41], cardiovascular disease, and mortality [42, 43] in CKD and hemodialysis patients, the beneficial effect of B-vitamin supplementation on lowering plasma homocysteine concentration in CKD and hemodialysis patients has not been confirmed [42, 43]. B-vitamin deficiency, increased oxidative stress, or other factors may be directly rather than indirectly (via elevated homocysteine levels) associated with acute or chronic inflammation; therefore, increased homocysteine concentrations and elevated inflammatory markers were not always associated with each other [24]. In the present study, vitamin B-6 status is independent of homocysteine, and being associated with inflammatory responses may support this hypothesis.

One limitation of this study was the use of a small number of stage 2–5 CKD patients as compared to the number of hemodialysis patients. The final recruitment number in the present study was larger than our sample size calculation; however, a greater number of patients may increase the statistical power to possibly reveal a more significant association between vitamin B-6 status and cytokine levels. Although we have excluded clinically unstable patients in the present study, cytokine levels in elderly adults have been reported to be varied day-to-day [46], while intraindividual variability of CKD and hemodialysis patients might be even greater than the variability in the general population. We did not measure the levels of other water soluble vitamins, such as folate and vitamin B-12, which might affect plasma homocysteine concentration and cytokine levels. Another limitation was that a cross-sectional design caused one time of blood test and therefore could not generally reflect long-term vitamin B-6, homocysteine, and inflammatory status. A longitudinal study seems necessary to better clarify the association of vitamin B-6 status, homocysteine, and inflammatory markers in CKD and hemodialysis patients.

## 5. Conclusions

Taken together, vitamin B-6 status rather than homocysteine seemed to be a significant factor in relation to inflammatory responses for CKD and hemodialysis patients. Further study regarding the association between homocysteine and inflammatory responses is warranted and must therefore be further investigated.

## Figures and Tables

**Table 1:** Demographic and clinical characteristics of patients with chronic kidney disease or those receiving hemodialysis.

Characteristics	Stage 2-3 CKD (*n* = 34)	Stage 4-5 CKD (*n* = 34)	Hemodialysis (*n* = 68)
Age (y)	52.76 ± 16.11^a^	57.53 ± 12.10^b^	61.56 ± 10.61^c^
Gender (Male/Female)	21/13	24/10	34/34
Waist circumference (cm)	87.96 ± 1.53^a^	88.67 ± 1.81^a^	82.93 ± 1.50^b^
Body mass index (kg/m^2^)	23.74 ± 3.63	25.17 ± 3.43	23.70 ± 3.91
Blood pressure (mmHg)			
Systolic	127.44 ± 2.26^a^	134.59 ± 2.27^a^	149.85 ± 3.23^b^
Diastolic	74.59 ± 1.41	76.41 ± 2.05	74.15 ± 1.97
Antihypertensive medication uses (*n*, %)	22, 64.71%	30, 11.81%	43, 63.23%
eGFR (mL/min/1.73 m^2^)	55.65^a^ ± 2.83	17.77 ± 1.06^b^	4.67 ± 0.17^c^
Serum creatinine (mg/dL)	1.38 ± 0.06^a^	3.84 ± 0.25^b^	11.13 ± 0.28^c^
BUN (mg/dL)	21.39 ± 1.10^a^	51.79 ± 2.81^b^	64.09 ± 1.90^c^
Serum albumin (g/dL)	4.40 ± 0.04^a^	4.15 ± 0.06^a^	3.82 ± 0.04^b^
<3.5 g/dL (*n*, %)	0	1, 0.03%	9, 13.24%
Serum glucose (mg/dL)	97.24 ± 4.91	114.06 ± 6.75	108.16 ± 4.71
Diabetes (*n*, %)	5 (14.71%)	13 (38.24%)	29 (42.65%)
Antidiabetic medication uses (*n*, %)	7, 20.59%	14, 41.18%	28, 41.18%
Total cholesterol (mg/dL)	187.12 ± 5.76^a^	169.41 ± 8.23^a,b^	160.71 ± 3.96^b^
Lipid lowering medication uses (*n*, %)	13, 38.24%	19, 55.88%	9, 13.24%
B-vitamin supplement uses (*n*, %)			
Yes	0	6 (17.65%)	33 (48.53%)
No	34, 100%	28, 82.35%	35, 51.47%
Current smoking habit (*n*, %)			
Yes	6, 17.65%	4, 11.76%	1, 1.47%
No	27, 79.41%	30, 88.24%	67, 98.53%
Current drinking habit (*n*, %)			
Yes	5, 14.71%	2, 5.88%	0
No	29, 85.29%	32, 94.12%	68, 100%

Values are means ± standard error. eGFR, estimated glomerular filtration rate; BUN, blood urea nitrogen; ^a,b,c^Values with a different superscript letter are significantly different among groups; *p* < 0.05.

**Table 2:** Vitamin B-6 status and inflammatory markers in patients with chronic kidney disease or those receiving hemodialysis.

	Stage 2-3 CKD (*n* = 34)	Stage 4-5 CKD (*n* = 34)	Hemodialysis (*n* = 68)
*Vitamin B-6 status*			
Pyridoxal 5′-phosphate (nmol/L)	58.50 ± 8.52^a^	84.31 ± 21.64^a,b^	148.16 ± 26.76^b^
<20 nmol/L (*n*, %)	3, 8.82%	5, 14.71%	2, 2.94%
Pyridoxal (nmol/L)	15.85 ± 1.71	22.03 ± 4.82	30.08 ± 4.37
Homocysteine (*μ*mol/L)	15.32 ± 0.65^a^	28.22 ± 1.57^b^	27.08 ± 1.03^b^
≥14 *μ*mol/L (*n*, %)	22, 64.71%	34, 100%	68, 100%
*Inflammatory markers*			
C-reactive protein (mg/L)	1.49 ± 0.52^a^	3.86 ± 1.00^b^	4.82 ± 1.17^b^
Interleukin-1*β* (pg/mL)	1.28 ± 0.58^a,b^	1.48 ± 0.18^a,c^	1.18 ± 0.31^b^
Interleukin-4 (pg/mL)	1.21 ± 0.30	0.97 ± 0.10	1.12 ± 0.17
Interleukin-6 (pg/mL)	10.38 ± 2.18	6.80 ± 0.97	6.36 ± 1.27
Interleukin-8 (pg/mL)	8.86 ± 1.31	7.73 ± 0.65	10.11 ± 1.59
Interleukin-10 (pg/mL)	13.76 ± 2.78^a^	3.92 ± 0.61^b^	6.35 ± 1.57^b^
TNF-*α* (pg/mL)	26.36 ± 8.35^a^	26.58 ± 5.42^b^	32.11 ± 13.50^a,b^

Values are means ± standard error. TNF-*α*, tumor necrosis factor-*α*; ^a,b,c^Values with a different superscript letter are significantly different among groups; *p* < 0.05.

**Table 3:** Partial Spearman's correlation coefficients (*r*_*s*_) of vitamin B-6 and homocysteine with inflammatory markers in all patients.

Parameters	*r*_*s*_ (no additionally adjusted homocysteine)			*r*_*s*_ (additionally adjusted homocysteine)		
	PLP (nmol/L)	PL (nmol/L)	PLP + PL (nmol/L)	PLP (nmol/L)	PL (nmol/L)	PLP + PL (nmol/L)
CRP (mg/L)	−0.22^*∗*^	—	−0.21^*∗*^	−0.22^*∗*^	—	−0.22^*∗*^
Interleukin-1*β* (pg/mL)	—^1^	—	0.22^*∗*^	—	—	0.22^*∗*^
Interleukin-4 (pg/mL)	—	—	—	—	—	—
Interleukin-6 (pg/mL)	—	—	—	—	—	—
Interleukin-8 (pg/mL)	—	—	—	—	—	—
Interleukin-10 (pg/mL)	—	−0.21^*∗*^	—	—	−0.23^*∗*^	—
TNF-*α* (pg/mL)	—	—	—	—	—	—

*N* = 136. Values are partial correlation coefficient (*r*_*s*_). ^*∗*^*p* < 0.05. Adjusting for age, gender, body mass index, systolic blood pressure, serum albumin, diabetes, chronic kidney disease stage, plasma homocysteine, B-vitamin supplement usage, smoking and drinking habits; PLP, pyridoxal 5′-phosphate; PL, pyridoxal; CRP, C-reactive protein; TNF-*α*, tumor necrosis factor-*α*; ^1^No significance.
